# Behavioural evidence of spectral opponent processing in the visual system of stomatopod crustaceans

**DOI:** 10.1242/jeb.247952

**Published:** 2025-01-08

**Authors:** Ching-Wen Judy Wang, N. Justin Marshall

**Affiliations:** Queensland Brain Institute, University of Queensland, St Lucia, QLD 4072, Australia

**Keywords:** Stomatopods, Colour vision, Spectral opponency, Invertebrate vision, Behaviour

## Abstract

Stomatopods, commonly known as mantis shrimps, possess intricate colour vision with up to 12 photoreceptor classes arranged in four specialised ommatidia rows (rows 1–4 in the midband region of the eye) for colour perception. Whereas 2–4 spectral sensitivities suffice for most visual systems, the function and mechanism behind stomatopods' 12-channel colour vision remains unclear. Previous anatomical and behavioural studies have suggested that binning and opponent processing mechanisms may coexist in stomatopod colour vision. However, direct evidence of colour opponency has been lacking. We hypothesised that if colour opponency exists in stomatopod vision, they would be able to distinguish colour from grey under coloured illumination. Conversely, if only the binning system is used, they would not. By examining the colour vision of the stomatopod *Haptosquilla trispinosa* with modified von Frisch grey card experiments, we found that they can differentiate between colour and grey under various coloured illuminations. Our results provide the first direct behavioural evidence of spectral opponency in stomatopods, suggesting that they use a hybrid colour processing system combining opponent and binning mechanisms for colour vision. This study advances our understanding of the complex visual system in stomatopods and highlights the importance of further research into the processing mechanisms, function and evolution of their unique visual system.

## Teaser

**Summary:** Our study provides the first behavioural evidence of spectral opponency in stomatopods, revealing their hybrid colour processing system, combining multi-opponent and binning colour processing mechanisms.

## INTRODUCTION

Stomatopods, also known as mantis shrimps, are marine crustacean predators inhabiting coral reef waters in tropical and subtropical environments. Having diverged from the other crustaceans approximately 400 million years ago, stomatopods have undergone an evolutionary path separate to other crustaceans, notably via the development of a sophisticated visual system (Manning, 1977; Marshall, 1988; Cronin and Marshall, 1989a,b; Marshall et al., 2007; Marshall and Arikawa, 2014). Their colour vision in particular is unique, utilising as many as 12 classes of photoreceptors to sample chromatic information from deep UV to far red (300–750 nm) (Marshall, 1988; Cronin and Marshall, 1989b; Thoen et al., 2017b). As most animals use 2–4 spectral sensitivities for colour vision, with more than four adding little discrimination ability (Barlow, 1982; Maloney, 1986), the reason for the apparent chromatic complexity of the stomatopod system remains unclear.

**Fig. 1.**
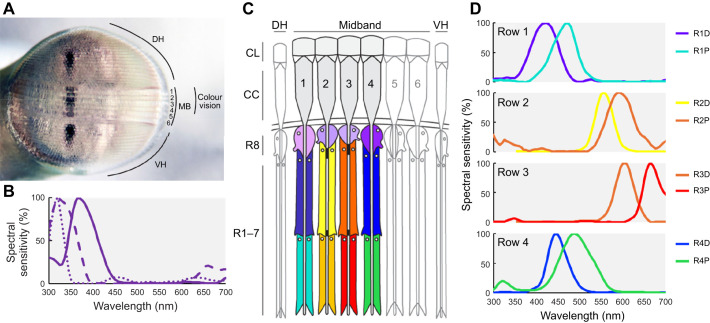
**Stomatopod eye structure and spectral sensitivities.** (A) Eye of *Haptosquilla trispinosa*, showing the dorsal/ventral hemispheres (DH/VH) and the six midband (MB) rows. Photo credit: N.J.M. (B) Spectral sensitivities of R8, adapted from Thoen et al. (2014). (C) Illustration of the sagittal section of midband rows 1–4, comprising three tiers with 12 spectral sensitivities. CL, corneal lens; CC, crystalline cone; DH, dorsal hemisphere; VH, ventral hemisphere. The illustration was adapted from Marshall et al. (2007). (D) Normalised spectral sensitivities of the two proximal tiers of R1–R7 in rows 1–4. D, distal; P, proximal. The graphs are adapted from Thoen et al. (2014, 2017b).

The compound eye of most shallow water stomatopods comprises dorsal and ventral hemispherical regions, separated by six rows of specialised ommatidia, known as the midband (Fig. 1A) (Marshall, 1988; Cronin and Marshall, 1989a; Porter et al., 2010). The dorsal four rows (rows 1–4) are specialised for colour vision, whereas the last two rows (rows 5 and 6) are dedicated to circular (or elliptical) polarisation vision at wavelengths ∼500 nm (Fig. 1A,C) (Cronin and Marshall, 1989a,b; Chiou et al., 2008; Thoen et al., 2017b). In addition to the midband, the hemispherical parts are employed for detecting linear polarisation and luminance information, exhibiting structures and functions similar to those in other crustaceans (Fig. 1A,C) (Cronin and Marshall, 1989a; Marshall et al., 1991; Kleinlogel et al., 2003; How and Marshall, 2014; Daly et al., 2016). Notably, the midband region possesses a higher spatial resolution compared with the hemispherical parts (Marshall and Land, 1993a). Stomatopods utilise this specialised midband to scan their visual environment, acquiring spectral and polarisation information in a serial fashion (Cronin and Marshall, 2001; Land et al., 1990). In the stomatopod eye, each ommatidium consists of eight retinular cells, termed R1–R8. The architecture of ommatidia varies depending on their function (Marshall, 1988; Cronin and Marshall, 1989b; Marshall et al., 1991, 2007; Marshall and Oberwinkler, 1999). Ommatidia responsible for polarisation vision demonstrate simpler structures, featuring two tiers, with a short R8 located distally to the main rhabdom consisting of R1–R7 (Fig. 1C). In contrast, the ommatidia designated for colour vision are divided into three tiers, including R8 and two separate tiers of R1–R7 (R1, R4 and R5 and R2, R3, R6 and R7). Each tier possesses a distinct spectral sensitivity, rendering the dorsal four rows of the midband an array of spectral channels, housing 12 classes of photoreceptors (Fig. 1B–D).

**Fig. 2.**
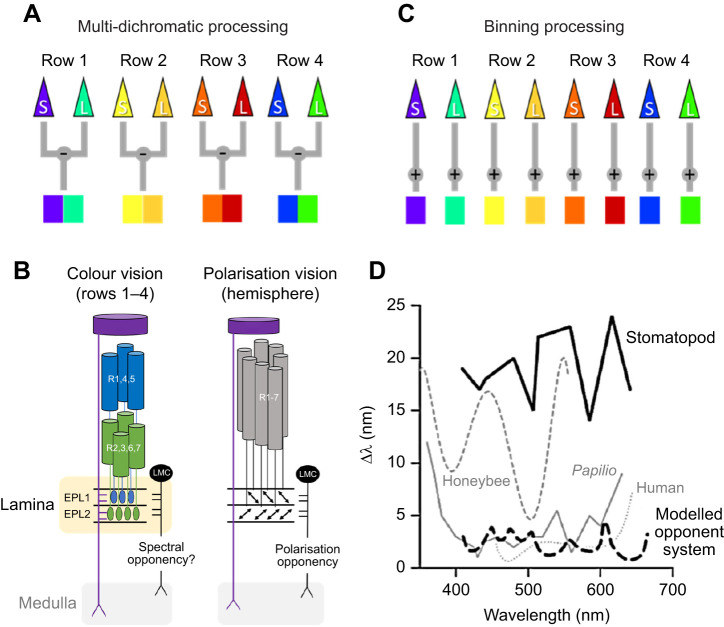
**Spectral discrimination curves and schematic diagrams of two processing hypotheses for stomatopod colour vision.** (A) Diagram of multi-dichromatic processing, illustrating opponent processing within each spectral-sensitive row. S: short-wavelength information; L: long-wavelength information. (B) The left diagram illustrates the proposed spectral opponent processing scheme of in midband rows 1–4, using row 4 as an example. The right diagram shows the polarisation opponent processing scheme in the hemispheres. EPL, external plexiform layer. (C) Binning processing scheme. (D) Spectral discrimination abilities of stomatopods and other animals, showing stomatopods have a coarse spectral resolution compared with the modelled results (black dashed line) and other animals. Figure is adapted from Thoen et al. (2014).

Previous behavioural studies by Marshall et al. (1996) using von Frisch grey card tests demonstrated that stomatopods possess true colour vision. However, the spectral processing mechanism stomatopods use in their polychromatic colour vision remained speculative. Two hypotheses regarding the processing mechanism of their colour vision have been proposed: (1) a multiple dichromatic colour-opponent system, and (2) a colour-binning or barcode system (Fig. 2). The multi-dichromatic processing hypothesis suggests that rows 1–4 function as four parallel dichromatic systems, comparing spectral information within each row to encode colour information in different spectral ranges; a schema generally similar to other colour vision systems (Fig. 2A) (Marshall, 1988; Cronin and Marshall, 1989b, 2001; Marshall and Land, 1993b; Marshall et al., 1996; Osorio et al., 1997). This hypothesis is based on the tiered arrangement of the R1–R7 cells in rows 1–4 and is supported by the neuronal organisation in the first optic lobe, the lamina ganglionaris. The axons of R1–R7 from each ommatidium project into individual processing units termed lamina cartridges, with the two subsets terminating at separate lamina layers (Strausfeld and Nassel, 1981; Kleinlogel et al., 2003; Kleinlogel and Marshall, 2005; Thoen et al., 2017a). Such neuronal construction closely resembles the polarisation opponent processing system used for comparing horizontally and vertically polarised signals in many crustaceans as well as the hemispheric regions of the stomatopod eye (Fig. 2B) (Strausfeld and Nassel, 1981; Marshall et al., 1991; Glantz and Miller, 2002; How and Marshall, 2014). Hence, it has been proposed that colour processing in stomatopods may have simply adopted this neuronal wiring to compare the different spectral signals perceived by each spectral row.

Conversely, the binning (barcode) processing system posits that colour perception in stomatopods relies on the activation pattern of the 12 channels, with the channel exhibiting the stronger signal determining the colour (Fig. 2C) (Thoen et al., 2014; Marshall and Arikawa, 2014). This hypothesis is supported by the unexpected low spectral resolution (Δ λ=15–25 nm) observed in wavelength discrimination tests (Fig. 2D) (Thoen et al., 2014), which is worse than other visual systems with fewer spectral receptors (Koshitaka et al., 2008; Thoen et al., 2014). For example, honeybee (Δ λ=5–20 nm; von Helversen, 1972), goldfish (Δ λ=3–20 nm; Neumeyer, 1986), human (Δ λ=1–8 nm; De Valois and Jacobs, 1968) and butterfly (Δ λ=<1–15 nm; Koshitaka et al., 2008) (see Koshitaka et al., 2008 for original diagram). Thoen et al. (2014) suggested that this binning system functions as a pattern analyser, similar to how the cochlea examines auditory space in the mammalian ear, allowing for efficient spectral recognition as opposed to fine discrimination in stomatopod colour vision (Thoen et al., 2014; Marshall and Arikawa, 2014).

However, while the concept of the binning system is intriguing for stomatopod spectral processing and is possibly the first of its kind, it does not necessarily exclude other processing methods. Streets et al. (2022) explored this by examining stomatopods' ability to distinguish unsaturated colours with grey stimuli. They hypothesised that if their colour vision relies solely on binning processing, low-saturated colours and grey would be indistinguishable, as they generate similar activation patterns across spectral channels. Interestingly, their findings showed that stomatopods can distinguish between these stimuli, suggesting that opponent processing may also play a role in their colour vision.

Building on these insights, our study aimed to address the lack of direct behavioural evidence for the colour-opponent system in stomatopod colour vision and to explore the multiple dichromatic mechanism theory. We conducted modified von Frisch grey card experiments (Von Frisch, 1914; Kelber et al., 2003; Streets et al., 2022) to examine the colour vision of the stomatopod *Haptosquilla trispinosa* under various coloured illuminations (blue, green and red). We hypothesised that if *H. trispinosa* employs spectral opponent processing, it should be able to distinguish colours from greys under coloured illumination. The results showed that *H. trispinosa* could indeed discriminate colour from greys under green and red illuminations but struggled under blue illumination. Using a multi-dichromatic model to compare the contrast between colour and grey stimuli, we found that the discrepancy may be due to the low contrast between blue and grey stimuli. Alternatively, this challenge could be related to the biological significance of blue signals in stomatopod courtship behaviour, which will be explored in the Discussion section. Overall, these findings suggest that stomatopods use a unique spectral processing system that combines both spectral opponency and binning for colour perception. This hybrid approach may explain their extensive range of spectral sensitivity coupled with relatively poor spectral discrimination, offering new insights into the visual processing and perception of the unique colour vision of stomatopods.

## MATERIALS AND METHODS

### Animal collection

Stomatopods, *Haptosquilla trispinosa* (Dana 1852), were collected from shallow reef areas on Lizard Island Research Station (LIRS) at a depth range of 0 to 5 m (GBRMPA permit no. G17/38160.1). Adult *H. trispinosa* typically measure between 2 and 3.5 cm in length. For this study, we used individuals ranging from 2.1 to 3.3 cm in length. Following collection, each individual was placed in a behavioural chamber (W×L×H:15×27×13 cm) for a period of 1–2 days for acclimation. Each chamber contained a grey PVC tube designed as a burrow with front and back lids. The front lid had a drilled hole serving as the burrow's entrance. To mimic their natural habitats, fresh sand sourced from their original environment was added to the chamber, filling it to approximately one-third of its depth to create a sandy base. The water level in each chamber was 5–7 cm from the surface to the sandy bottom. To avoid degradation of their colour vision, the experiments were performed only on freshly caught stomatopods.

This study comprised two separate experiments: the first was conducted from 19 July to 12 August 2021 and the second from 19 February to 5 March 2022. Both experiments took place at the outdoor bench area at LIRS, sheltered beneath clear polycarbonate roofs. While some UV is blocked from above by this roofing, UV still reaches the experimental arena from the sides. As the experiments described here are not in UV wavelengths, this slight reduction was assumed to be irrelevant.

### Stimulus and illumination design

**Fig. 3.**
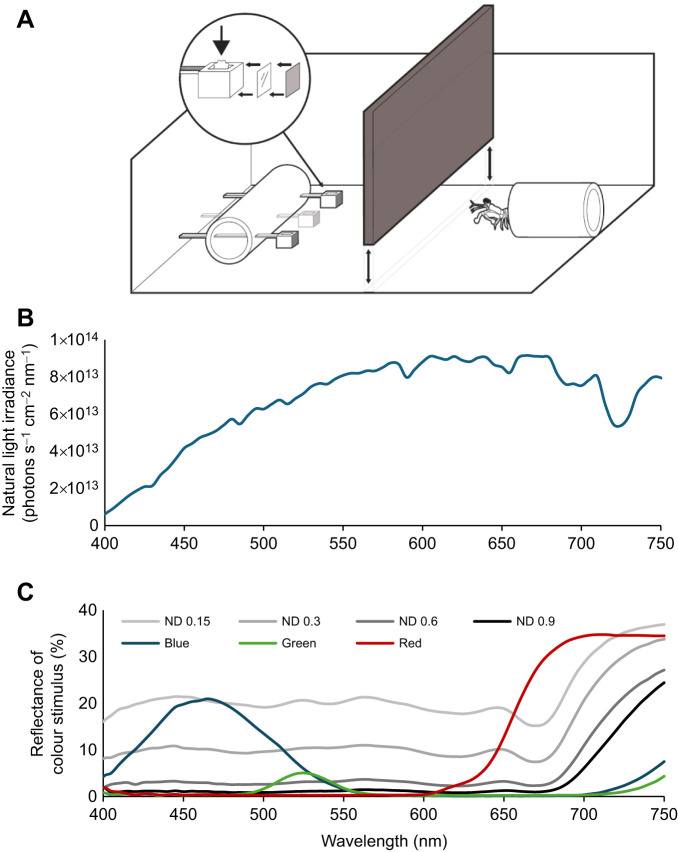
**Experiment design of the grey card experiment.** (A) Diagram adapted from Streets et al. (2022), showing the behavioural chamber for colour discrimination tests. During training, food reward (indicated by the arrow) was placed inside the camber of the target cable ties. (B) Irradiance spectrum of the natural light illumination at Lizard Island Research Station, measured at where the experiments were conducted. (C) Reflectance spectra of colour and grey stimuli, measured from the surface of cable ties.

A modified von Frisch grey card experiment (Fig. 3A) (Von Frisch, 1914; Kelber et al., 2003; Templin, 2017; Streets et al., 2022) was employed in this study to investigate stomatopods' ability to distinguish colour from greys under natural light and various illumination conditions. White plastic cable ties (Crescent, 2.5 mm width, 10 cm long) were used to present different stimuli to stomatopods. The size of the cable tie head, measuring 4×5×4 mm (W×L×H), was specifically chosen to mimic appropriate prey size for *H. trispinosa*, allowing easy grasping and transportation back to their burrows. Each cable tie was fitted with either a colour filter (for targets) or a neutral density (ND) filter (for distractors), each measuring 4×4 mm, on its front surface using white double-sided tape.

**Fig. 4.**
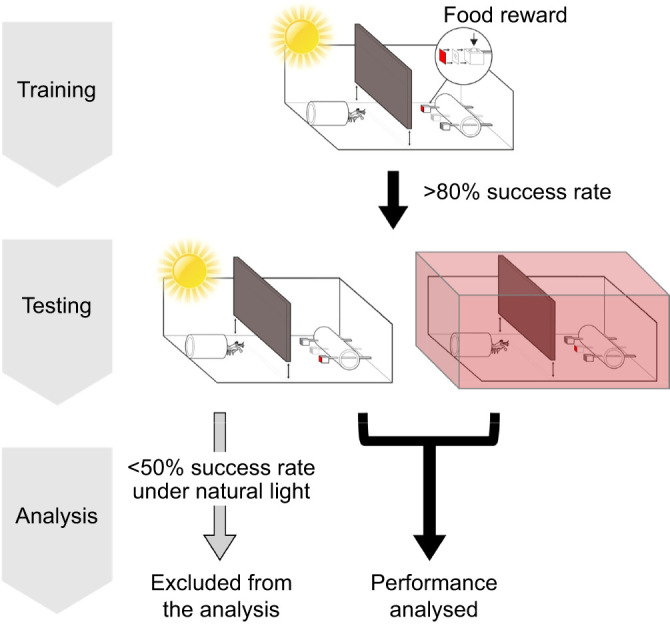
**Procedure of the colour discrimination ability experiment.** During training, food reward was placed in the colour cable tie while the grey cable ties remained empty. Animals that passed 80% success rate for 5 consecutive trials were moved to the testing stage. At the testing stage, stomatopod colour discrimination abilities were tested under natural light and coloured illumination. Individuals with a success rate lower than 50% under natural light were excluded from the experiment as this indicated that they failed to associate the reward with visual stimuli. For those individuals that were able to maintain high performance under natural light, their performance under coloured illumination was analysed to examine colour discrimination abilities under coloured illuminations.

Throughout the experiment, each animal was assigned to one of three colour targets: blue, green or red, which are referred to as the blue, green and red groups. During both training and testing trials, three stimuli were presented to the animals in each colour group: one colour target and two grey distractors. The grey distractors were selected from four types of grey stimuli made with 0.15, 0.3, 0.6 and 0.9 ND filters, resulting in six combinations of the distractor intensities for tests: 0.15/0.3, 0.15/0.6, 0.15/0.9, 0.3/0.6, 0.3/0.9 and 0.6/0.9 (ND). The position and combination of the stimuli were arranged using a pseudo-randomised method, with adjustments made to avoid repetitive colour or grey stimuli occurring at the same position for more than two consecutive trials. The stimulus combinations and test order are listed in Table S1, and an illustration of the experimental setup is shown in Fig. 4. To associate food rewards with correct choices, a small piece of shrimp meat was placed exclusively in the ratchet end cavity of the colour cable ties (targets), while the grey cable ties (distractors) remained empty and uncontaminated by food. Customised cable tie holders, crafted from white PVC tubes perforated with three holes aligning horizontally at each side, were utilised to display the cable ties with consistent distance and height throughout the experiments. The distance between each stimulus was approximately 1.5 cm, and the stimuli were initially placed 5 to 8 cm from the animals, which was slightly longer than their body length. This initial placement encouraged the animals to fully exit their burrows when making a choice. However, it is important to note that our experimental setup allowed animals to view the targets from various distances and angles, as the animals were free to approach and examine the stimuli closely before making a decision.

**Fig. 5.**
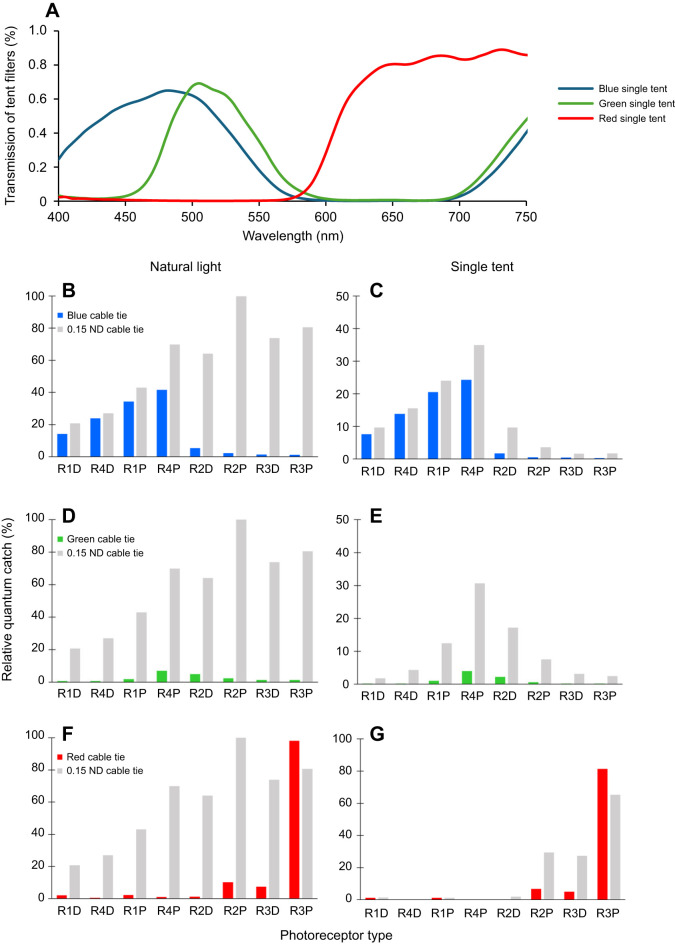
**Transmittance of the tent filters of experiment 1 (single-layered tent) and the relative photon catch for each photoreceptor under natural light and coloured illuminations.** (A) Transmittance of the filters used to change the illumination. (B–G) Normalised quantum catch of the receptors when viewing blue, green or red, and grey (ND 0.15) stimuli under natural light (B,D,F) and under corresponding coloured illumination (C,E,G). Photoreceptor types are arranged according to the spectral sensitivities of receptors, numbers indicate the row number of rows 1–4; D, distal; P, proximal. All photons catch values were normalised to the highest photon catch value observed from R2P when stimulated by grey (ND 0.15) under natural light.

**Fig. 6.**
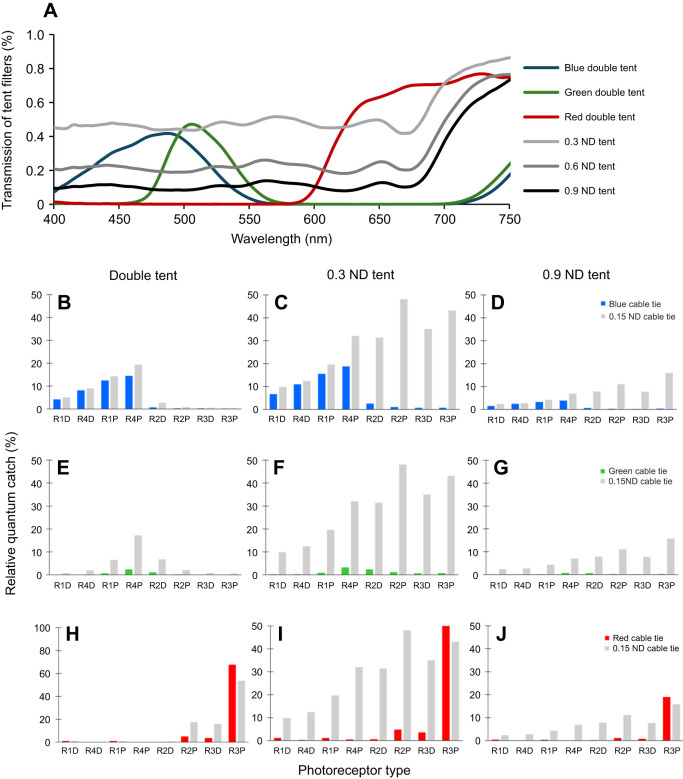
**Transmittance of the tent filters of experiment 2 (double-layered and grey tents) and the relative photon catch for each photoreceptor under different illuminations.** (A) Transmittance of the filters used to change the illumination. (B–J) Normalised quantum catch of the receptors when viewing blue, green or red, and grey (ND 0.15) stimuli under double-layered corresponding coloured illumination (B,E,H) and 0.3 ND filter tent (C,F,I) or 0.9 ND filter tent (D,G,J). Note that there were 3 ND filter tents used to modify the natural light intensity; here, we show the quantum catch of only the 0.3 and 0.9 ND filters as examples. All photon catch values were normalized to the highest photon catch value observed from R2P when stimulated by grey (ND 0.15) under natural light.

The spectral measurements were conducted using an Ocean Optics USB 2000 spectrophotometer. Downwelling light at the experiment site was measured within the behavioural chamber underwater (Fig. 3B). The reflectance of stimuli (Fig. 3C) and filter transmission (Figs 5A and 6A) were measured in air under controlled lighting conditions. Fig. 3C illustrates the reflectance spectra of the various colour and grey stimuli. The colour filters used to make targets included blue (≈360–550 nm; LEE Filters #183), green (≈500–560 nm; LEE Filters #139) and red (>610 nm; LEE Filters #027). Grey distractors were created using 0.15 ND (LEE Filters #209), 0.3 ND (LEE Filters #209), 0.6 ND (LEE Filters #210) and 0.9 ND (LEE Filters #211), where higher ND values indicate greater light attenuation.

In terms of the light conditions, the first experiment was conducted under natural light and coloured illuminations, blue, green and red lights. To alter the lighting within each behavioural chamber, customised filter tents were crafted from plastic sheets and applied during coloured illumination tests. The transmittance spectra of these filters are shown in Figs 5A and 6A. The coloured filters included blue (≈360–590 nm; LEE Filters #172), green (≈450–600 nm; LEE Filters #124) and red (>590 nm; LEE Filters #182). Each coloured illumination was applied only to animals trained with the corresponding coloured targets: blue illumination for blue-trained, green for green-trained and red for red-trained animals.

The second experiment aimed to test colour vision under dimmer natural light and coloured illuminations. To create dimmer coloured lighting, double-layered colour filters were used to reduce the light intensity. For dimmer natural light conditions, ND filters with varying optical densities, including 0.3 ND (LEE Filters #209), 0.6 ND (LEE Filters #210) and 0.9 ND (LEE Filters #211), were employed to attenuate natural light.

### Experiment design

An established choice test procedure outlined by Templin (2017) and Streets et al. (2022) was followed to train and test stomatopod *H. trispinosa*. The experiment comprised three stages: priming, training and testing. Before priming, the animals were acclimatised for 1–2 days without food to ensure a high level of hunger, facilitating quicker associations between choice and food reward. During each experiment stage, animals underwent three trials per day: one each in the morning, at midday and in the afternoon, with each trial consisting of a single test.

#### Priming (2–3 days)

During the priming stage, animals were introduced to a single cable tie of their assigned colour. The cable tie contained food in the ratchet cavity and was placed at one of the three positions (left, middle and right) in the holder. The position of the cable tie varied for different trials with a random order. In each priming trial, animals were given 5 min to grasp the cable tie before the trial was aborted. After the trial, cable ties were retrieved, cleaned with fresh water and recycled for future trials. All animals underwent priming trials three times. Individuals that reached an 80% participation rate in the last 5 consecutive trials were allowed to proceed to the training stage. Animals with participation rates lower than 50% were considered unmotivated, potentially due to stress, sickness or moulting. Therefore, these animals were deemed untrainable and replaced with new animals.

#### Training (3–5 days)

During the training stage, animals were presented with three stimuli in each trial including one colour stimulus (target) and two grey stimuli (distractors) (Fig. 4, training). Only the colour stimulus contained a food reward in the cable tie, while the distractors remained empty and odourless. Animals were given 5 min for each training trial. A correct choice was recorded if their first selection was the colour cable tie. Conversely, it was considered wrong choice if they chose a grey cable tie. Regardless of their choice, animals were permitted to explore any other cable ties within the 5 min time frame.

All training trials were conducted three times a day under natural light. The performance of each individual was assessed after 3 days of training (9 trials). Individuals with over 80% success rate (correct choice) in the last six consecutive trials were allowed to proceed to the testing stage. Individuals with success rate between 30 and 80% were kept in the training group for more training trials. Those with success rates below 30% were considered untrainable and therefore removed from the experiment.

#### Testing (10–16 days)

In the testing phase, all colour target and ND distractor stimulus were constructed with new cable ties, carefully kept separate from the training cable ties to avoid any food and therefore olfactory contamination. Similarly to the training trials, one colour target and two grey distractors were presented in each testing trial. The combination of the stimulus was also randomised with adjustments to avoid recurrence of the same combination set or target positions. Three trials were conducted each day with each trial was conducted under either natural light or coloured illumination conditions (Fig. 4, testing; Movie 1). To alter the lighting throughout the experiment, a colour tent was applied to cover the behavioural chamber shortly before each trial. After setting up the tent, stimuli were introduced into the chamber by slightly lifting the tent, with the experimenter's fingers covering the stimuli during transport. This setup process took approximately 30 s, which was the duration the animals were exposed to coloured illumination before each trial. The tent was then removed immediately after the trial to ensure the animals' visual systems were exposed to natural light between trials.

The testing phase commenced with natural light tests on the first day and coloured illumination tests were introduced from the second day onwards. This gradual transition was to allow individuals to accustomed to the testing procedure. Starting from the second day of testing, one natural light and two colour illumination tests were performed in random order each day for every individual.

In each testing trial, the individuals had up to 5 min to make a choice in each trial, with the trial ended once a choice was made. A correct choice was marked when the individual grabbed or struck the colour cable tie. After a correct choice was made, a food reward would be delivered to the individual with forceps. However, if the individual made an incorrect choice, the cable ties would be retrieved and no food would be given to the animal. Similarly, if the individual did not make any choice during the time frame, the cable ties would be removed and the individual would not receive a food reward.

Additionally, one reinforcement trial was conducted after every six testing trials. The procedure for the reinforcement trials followed that of the training trials, with food rewards provided using cable ties, allowing individuals to explore all options within the designated time frame. Choices made during reinforcement trials were excluded from the data analysis.

### Statistical analysis

The purpose of the analysis was to compare stomatopod colour discrimination abilities under both natural light and coloured illumination and determine whether their performances under the two conditions differ. After the individuals completed each testing trial, the overall performance of each individual was carefully examined before analysis. The colour illumination tests aimed to assess colour discrimination abilities under a restricted spectrum (blue, green and red), while natural light tests were conducted simultaneously to evaluate the reliability of each individual's performance. With a 33% probability of randomly choosing the colour stimulus, a threshold of a 50% success rate was established to determine whether an individual was well-trained and capable of maintaining their performance throughout the testing phase (Fig. 4, analysis). To ensure the reliability of an individual’s performance, only animals demonstrating a success rate higher than 50% under natural light conditions were included in the data analysis. Individuals with poor performance (below 50%) under natural light conditions were considered to have failed in training, possibly due to factors such as forgetting tasks or experiencing physical issues like stress, sickness, or moulting. In such cases, their performance under coloured illumination was regarded as unreliable and subsequently excluded from the analysis.

All analyses were performed in R studio (v. 4.1.2; r-project.org). Chi-square independence tests were employed to evaluate colour discrimination ability, comparing observed and expected counts of choices in three-choice tests with one target and two distractors. Therefore, expected counts for correct choices=total counts×⅓ and expected counts for incorrect choices=total counts×⅔.

Additionally, generalized linear mixed models (GLMMs) were executed using the lme4 package (CRAN.R-project.org/package=lme4) to explore the relationship between various factors (individuals, illuminations conditions or target positions) and choice outcomes. The model, applying a binomial distribution with a ‘logit’ link function, considered animal choices during tests as the variable of interest. Individual ID and sex were treated as random effects since there were multiple tests per individual, while illumination conditions, distractor combination and position of choice were considered fixed effects. However, sex, not significantly contributing to explanatory power, was excluded from the final models.

### Quantum catch of different photoreceptor types

When examining different stimuli under various illuminations, the activation levels of photoreceptors were quantified using the formula:
(1)

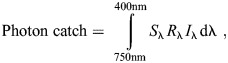
where *S*_λ_ represents the spectral sensitivity of the receptors of *H. trispinosa*, determined by the intracellular recording results (Thoen et al., 2017b); *R*_λ_ is the reflectance of the stimuli; *I*_λ_ is the photon number distribution of the illumination and λ denotes the wavelength. Fig. 5B–G illustrate the photon catches of receptors activated under different conditions in experiment 1, while Fig. 6B–J shows the photon catches of receptors in experiment 2.

### Measurement of contrast differences

To explore whether the contrast difference between colour and grey stimuli contributed to stomatopods' poor performance with the blue stimulus, we applied the multi-dichromatic processing model proposed by Marshall et al. (1996). This model, similar to the polarisation processing system described by How and Marshall (2014), compares spectral signals instead of polarisation signals. It is presumed that the spectral signals from the two receptor subsets (R1, R4, R5 and R2, R3, R6, R7) within the R1–R7 are compared in this process through the interneurons (Fig. 2B, colour vision). Accordingly, the output of the interneuron decoding a stimulus *X* in a row *n* is referred to as the activity profile (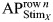), represented by the equation:
(2)

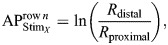
where, *R*_distal_ and *R*_proximal_ represent the quantum catches of the two receptor subsets in row *n* (one of the rows 1–4), with their receptor potential approximated by the natural log of the quantum catch. Each row *n* produces a distinct activity profile for a given stimulus. The contrast difference (CD) between two stimuli, denoted as stimulus *X* (Stim*_X_*) and stimulus *Y* (Stim*_Y_*), is calculated by subtracting the activity profiles generated for each stimulus in the same row *n*:
(3)

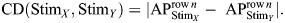


To determine whether the contrast difference of colour and grey stimuli to stomatopod colour vision aligns with the behavioural results, we measured the contrast differences between the stimuli for each row of rows 1–4 under natural light and coloured illuminations.

## RESULTS

The experiments tested the ability of stomatopods to discriminate between colour and grey stimuli under coloured illumination. Each animal was trained and tested with specific colour target and corresponding illumination: blue, green or red. For example, animals trained with the red target were tested under red illumination and were referred to as the red group, and the same applies to the other groups.

### Experiment 1 – colour discrimination ability under colour illumination

**Fig. 7.**
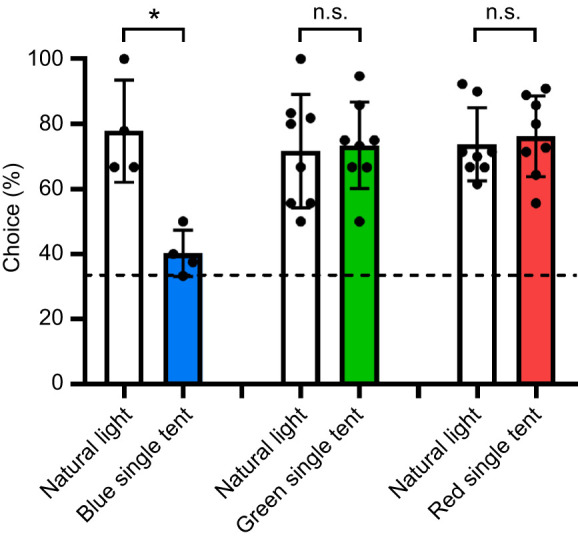
**Colour discrimination abilitie*s* of *Haptosquilla trispinosa* under natural light and different coloured illumination.** Histogram shows the success rates of blue-, green- and red-trained *H. trispinosa* when tested under natural light and coloured illumination. Each dot indicates the average performance of an individual. Dashed line represents the random choice probability (33.3%) from the three-choice test. Values are means±s.e.m. *P<0.05, n.s., not significant.

In experiment 1, a total of 69 stomatopods were trained, including 27 in the blue group, 20 in the green group, and 22 in the red group. Among these, 20 exhibited reliable performance under natural light conditions throughout testing, comprising 4 in the blue group, 8 in the green group and 8 in the red group. Fig. 7 illustrates the success rates under natural light and coloured illuminations. For the blue group, individuals displayed proficiency in identifying blue stimuli from greys under natural light with a success rate of 76% (*N*_individuals_=4; *N*_trials_=25). However, their performance declined to 40% when tested under blue illumination (*N*_individuals_=4; *N*_trials_=20). Statistically, it was not significantly different from the random choice rate of 33% (Chi-square test: *P*=0.3291). Moreover, GLMM analysis indicated a significant decrease in their correct choice rate under blue tents compared with natural light conditions (GLMM, *P*<0.05), suggesting that blue-trained animals struggled to distinguish blue stimuli from distractors under blue illumination.

In contrast, individuals in the green and red groups showed the ability to distinguish colours from greys even under coloured illuminations. The green group, with a success rate of 71.08% under natural light (*N*_individuals_=8; *N*_trials_=83), maintained a high success rate of 75.7% under green illumination (*N*_individuals_=8; *N*_trials_=107). Similarly, the red group, with a 74.73% success rate under natural light (*N*_individuals_=8; *N*_trials_=91), sustained a success rate of 73.74% under red illuminations (*N*_individuals_=8; *N*_trials_=99).

These choice rates significantly deviated from the probability of random choices (Chi-square test: green, *P*<0.0001; red, *P*<0.0001) and did not significantly differ from the performance tested under natural light (GLMM, *P*_green_=0.5205; *P*_red_=0.956). These findings indicate a consistent ability to discern colours from greys, irrespective of changes in illumination, emphasizing their capability to conduct colour vision with only 2–3 spectral receptors operating under restricted spectra.

### Experiment 2 – colour discrimination ability under dimmer illuminations

**Fig. 8.**
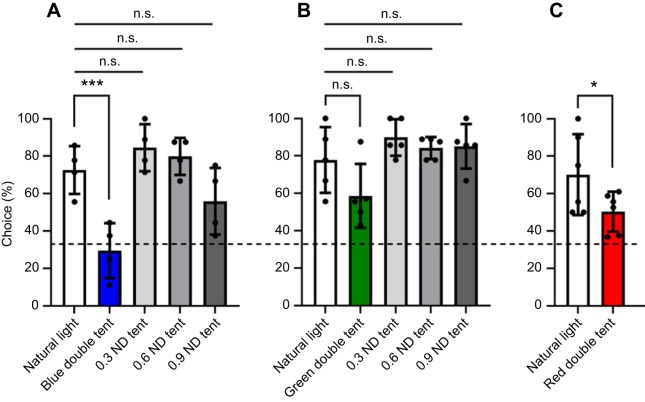
**Colour discrimination abilities of *H. trispinosa* under natural light and different dim illuminations.** (A) Performance comparison under double-layered blue tent and varying natural light intensities. (B) Performance comparison under double-layered green tent and varying natural light intensities. (C) Performance comparison under double-layered red tent and natural light. Each dot indicates the average performance of an individual. Dashed line represents the random choice probability (33.3%) from the three-choice test. *P<0.05, ***P<0.001; n.s., not significant. Values are means±s.e.m.

In this experiment, 40 individuals were primed and trained. Only 15 showed reliable performance under natural light tests and were included in the data analysis, consisting of 4 individuals from the blue group, 6 from the red group and 5 from the green group. For the blue and red groups, stomatopod colour discrimination ability declined under dim coloured illumination compared with their performance under natural light (Fig. 8). The blue group, which could discriminate blue from greys with a 71.86% success rate under natural light (*N*_individuals_=4; *N*_trials_=32), experienced a significant drop to 29.41% under the double-layer blue tent (*N*_individuals_=4; *N*_trials_=34). This success rate was significantly lower than that under natural light (GLMM, *P*<0.0001) and not significantly different from random choice (33.3%) (Chi-square test: *P*=0.9403), indicating their inability to discriminate blue targets from greys under dimmer blue illumination.

Similarly, the red group exhibited a decline in performance under dimmer red light. While having a success rate of 68.97% under natural light (*N*_individuals_=6; *N*_trials_=58), their success rate reduced to 50.70% under double-layer red tent (*N*_individuals_=6; *N*_trials_=71). Although this success rate was significantly different from random choice probabilities (33.3%) (Chi-square test: *P*<0.001), it was significantly lower than their performance under natural light condition (GLMM, *P*<0.05).

However, the green group demonstrated a high success rate under dim-coloured illumination. There was no significant difference between the success rate under natural light (78.57%, *N*_individuals_=5; *N*_trials_=42) and double-layer green tent (58.97%, *N*_individuals_=5; *N*_trials_=39) (GLMM, *P*=0.6).

In the ND tent tests, blue and green groups showed exhibit similar, achieving high success rates under varying natural light intensities. For the blue group, the success rates under 0.3 ND, 0.6 ND and 0.9 ND tents were 84.38% (*N*_trials_=32), 79.41% (*N*_trials_=34), and 55.17% (*N*_trials_=29), respectively. No significant differences were found between the success rates under natural light and any ND tent (GLMM, p_ND0.3_=0.22, p_ND0.6_=0.46, p_ND0.9_=0.20).

Likewise, the green group maintained a high success rate of over 80%, even with the dimmest illumination. The success rates under different ND tents were 89.19% for the 0.3 ND tent (*N*_trials_=37), 84.09% for the 0.6 ND tent (*N*_trials_=44) and 86.84% for the 0.9 ND tent (*N*_trials_=38). No significant differences in their success rates between natural light and any ND tent treatments in the green group (GLMM, *P*_ND0.3_=0.21, *P*_ND0.6_=0.51, *P*_ND0.9_=0.34).

It is important to note that no ND tent treatments were applied to the red group owing to the non-neutral filtering property of the ND filters in the long wavelength range. Although these filters exhibit effective neutral filtering effects in the short- and medium-wavelength ranges, they are unable to attenuate light effectively in the red spectral range (over 680 nm), making them unsuitable for the purpose of the test (Figs 1D and 6A). Therefore, the ND tent test for the red group was aborted.

Additionally, the distractor combination was included as a fixed effect in the GLMM analysis. Most combinations, across various light conditions, showed no significant impact on choice, suggesting that the combinations themselves did not influence the animals' decisions. However, an exception was observed with the red target under the red double tent condition in experiment 2. In this case, two specific combinations (0.15/0.9 and 0.3/0.6) significantly affected the animals' performance, with a tendency for them to select the darker grey distractor. Detailed results regarding the combination effects are presented in Tables S2 and S3.

### Behavioural variances with blue stimuli and contrast between stimuli

Interestingly, the blue group not only failed in colour discrimination under blue illumination tests, but also exhibited distinct behaviour compared with the other two groups. In experiment 1, more individuals from the blue group were required for training to reach the threshold of over 8 individuals needed for testing. While all groups performed well under natural light, the blue group's correct choice rate was 53.6% (*N*_individuals_=9, *N*_trials_=69), significantly lower than the green (72.72%, *N*_individuals_=9, *N*_trials_=88) and red groups (74.73%, *N*_individuals_=8, *N*_trials_=91). Additionally, five individuals from the blue group were excluded owing to poor performance (correct choice rate under 50% under natural light), compared with one from the green group and none from the red group. This led to a much lower trainability for the blue group (14.81%) compared with the green (40%) and red groups (36.36%). Lastly, the blue group also had three times more no-choice instances during testing. These findings suggest that blue was a more challenging cue to learn than the other two colours. For more details of the performances of each group, see Table S4.

**Fig. 9.**
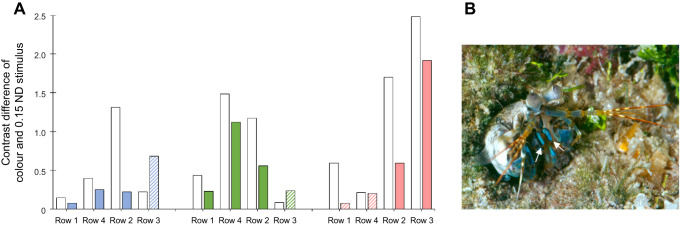
**Potential factors contributing to behavioural variance of *H. trispinosa* with blue stimuli.** (A) Contrast difference (CD) between colour and 0.15 ND grey stimuli as determined by spectral rows 1–4 under both natural light (white bars) and coloured illuminations. The filled colour bar indicates the activated spectral row, whereas the hatched bars represent the rows that were not activated under the corresponding coloured illumination. (B) Image of *H. trispinosa*, showing blue reflection of the maxillipeds (arrows) and other body parts.

In analysing the contrast differences (CDs) between colour and grey stimuli, we found that contrast was generally higher under natural light than under coloured illuminations. Fig. 9A illustrates the CD of the colour stimuli and 1.5 ND as an example, with additional comparison of CD values of colour and other grey stimuli shown in Fig. S1. It is interesting to note that both the green and red groups had two-three rows with high CD (>0.5) under natural and coloured illumination, whereas the blue group had only one row with high CD under natural light. Under coloured illuminations, the contrasts between green or red stimuli and grey remained high across multiple rows, while the contrast between blue and grey stimuli was extremely low contrast profile (<0.5) across all rows, likely accounting for their poor performance under blue illumination tests.

It is noteworthy that although row 3 seemed to present a relatively high CD under blue illumination, it contains only long-wavelength receptors, meaning the signals perceived from row 3 under blue light would be extremely limited for effective signal processing. Similarly, row 3 in the green tests and rows 1 and 4 in the red tests were likely not effectively activated under the coloured illuminations, rendering their CD values unhelpful for colour identification.

## DISCUSSION

### Behavioural evidence of spectral processing in stomatopods' colour vision

Our findings reveal that the stomatopod *H. trispinosa* can discriminate colour from grey stimuli in the green and red spectral windows, although not under blue. The high success rates under green and red illumination provide compelling evidence that stomatopods can achieve colour vision using only a few rows in the midband rows 1–4. This provides the first direct behavioural evidence of opponent processing in stomatopod colour vision, as distinguishing colour and grey stimuli under coloured illumination would be implausible with the use of binning system alone. This result supports the hypothesis proposed by Streets et al. (2022) that opponent and binning processing systems may coexist in stomatopod colour vision. This hybrid system may explain the lack of fine spectral discrimination observed in Thoen et al. (2014), while still incorporating some opponent processing in their visual processing. Additionally, spectral opponency is supported by both the neuronal architecture of stomatopod colour vision and the theory that it evolved to reduce flicker in shallow water environments (Maximov, 2000). This adaptation might be particularly beneficial for stomatopods, as many of them inhabit shallow waters where reducing flicker can improve prey detection and threat response.

Additionally, these findings raise the question of whether each row in rows 1–4 can operate independently for colour vision. We revealed that stomatopods are able to perceive colour under different illuminations with activation of only 2–3 rows, implying the processing between different rows can be somewhat independent. Ideally, to understand opponent processing within a specific row, one would need to isolate activation to that row. However, none of the illuminations used managed to restrict activation to a single row owing to the overlapping or intertwined spectral sensitivities of different rows (Thoen et al., 2017b). Notably, red illumination, which activated only row 3 fully, still allowed colour perception, suggesting that single-row processing might be sufficient for colour discrimination. Given the similar construction of rows 1–4, it is likely that each row can function as an independent processing channel, supporting the hypothesis of multi-dichromatic processing (Marshall, 1988; Cronin and Marshall, 2001; Marshall et al., 1996; Osorio et al., 1997). However, it is important to note that the presence of cross-row opponency remains unclear, and further studies on neuron connectivity and electrophysiological investigations are needed for a more detailed understanding.

**Fig. 10.**
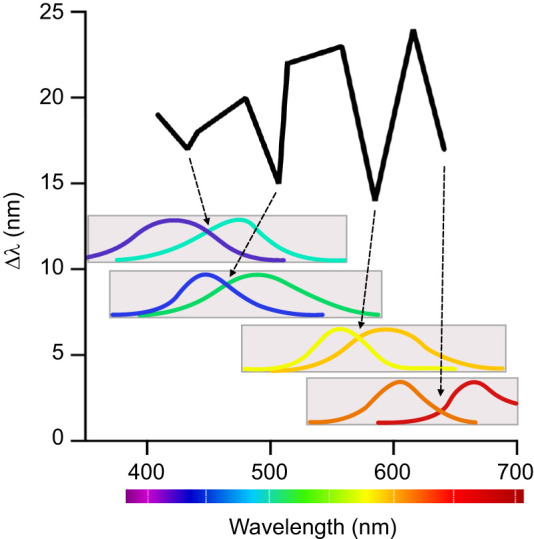
**Spectral discrimination curve alignment with the multi-dichromatic processing system in *H. trispinosa*.** The spectral discrimination curve (Δ λ) observed in *H. trispinosa* (top) (Thoen et al., 2014), showing four distinct minima that indicate enhanced spectral resolution in these animals. Spectral sensitivities of the multi-dichromatic pairs in rows 1–4 show the alignment of the minima with the sampling range of each row.

Furthermore, the presence of multi-dichromatic processing may explain the pattern of the spectral sensitivity curves of *H. trispinosa* demonstrated by Thoen et al. (2014). Spectral discrimination curves typically show minima between spectral sensitivity curves, with the number of minima reflecting the dimensions of colour vision – dichromats have one, trichromats two and tetrachromats three. Thoen et al. (2014) identified four minima in the spectral discrimination curve (Δ λ) within the 400–750 nm range in stomatopod colour vision (Fig. 10A), despite the presence of eight spectral sensitivities in R1–R7 across rows 1–4 (excluding UV-sensitive R8 cells). This indicated that stomatopods use an unconventional processing system compared to colour vision of other animals. Interestingly, the alignment between these four minima and the spectral sampling windows of rows 1–4 offers the possibility that the minima may result from the multi-dichromatic processing in their colour vision (Fig. 10B).

In experiment 2, we investigated the colour discrimination ability of stomatopod *H. trispinosa* under dim natural light and coloured illumination. The dimmest natural light condition (0.9 ND) resulted in the lowest photon catch for their visual system, lower than double-layered colour tents. Despite having a higher overall photon catch level, colour discrimination ability under dim coloured illuminations declined more than that under dim natural light. This implies that while rows 1–4 may function as a multi-dichromatic system capable of colour vision within a single row, their full capacity might still be more effective for colour discrimination, particularly in low light. However, it remains unclear whether this advantage is attributed to the activation of all spectral rows or the incorporation of luminance information. Future studies with controlled brightness are needed to understand the role of light intensity in colour discrimination under various spectral conditions.

Our experimental design with coloured tents effectively altered the hue of the grey stimuli according to the illumination. This means that the spectral cues of grey and colour stimuli under coloured illuminations could be very similar, requiring spectral opponency for discrimination. It is indeed possible that the colour and specific grey stimuli had matching spectral information under coloured illuminations, potentially contributing to some mistakes in identification. However, our GLMM analysis revealed that most combinations of distractors did not significantly affect the stomatopods' choices. Only two specific combinations (0.15/0.9 and 0.3/0.6) demonstrated a significant effect on performance, with animals tending to choose the darker grey distractor. This indicates that 0.9 and 0.6 ND might be indistinguishable from the red target under dim red illumination, leading to more incorrect choices. Nevertheless, in most light conditions and combinations, stomatopods successfully distinguished between the trained colour and different grey distractors with colour hue.

These results hint at the possibility that stomatopods possess colour constancy: the ability to perceive consistent colours under varying lighting conditions, in this case, the trained colour targets. However, there is currently no published behavioural evidence demonstrating colour constancy in stomatopod colour vision. A previous visual modelling study suggested that multi-dichromatic colour vision may be an adaptation for enhancing colour constancy in the aquatic environment (Osorio et al., 1997). It is suggested that the narrow spectral sensitivities and independent processing channels in stomatopods likely enable them to have exceptional colour constancy. Future studies on stomatopods' colour constancy will provide valuable insights into their unique colour vision system and its adaptive significance.

In addition to colour constancy, factors such as the distance between stimuli may also impact their discriminability of stimuli in stomatopod vision. In our study, we maintained a distance of approximately 1.5 cm between stimuli, consistent with prior research by Streets et al. (2022). However, our understanding of *H. trispinosa* visual acuity and image formation in stomatopods remains limited, particularly given their midband eye design and dynamic eye movements (Land et al., 1990). Future research on these visual features should provide a valuable foundation for studying how inter-stimulus distance may affect discriminability in stomatopod vision.

### Deciphering the discrepancy: behavioural variances with blue stimuli

Despite successful colour discrimination in green and red light, *H. trispinosa* struggled under blue illumination, which covered a broad spectrum and activated many photoreceptors. This unexpected result suggests two possible explanations for the discrepancy.

#### Potential low contrast between blue and grey stimuli

One possible explanation is that the contrast between blue and grey stimuli was lower than that of green or red with grey. This is supported by the finding that both the green and red groups had more rows with high CD under natural light and coloured illumination than the blue group (Fig. 9A, white bars), likely contributing to the blue group's lower trainability across all experiments. Additionally, while the contrast between blue and grey stimuli was sufficient for identification under natural light, the extremely low contrast profile across all rows under blue illumination provides a convincing explanation for their failure under blue illumination tests (Fig. 9A, coloured bars).

Overall, the contrast differences calculated with the multi-dichromatic model support the observed behavioural discrepancy, showing that the poor performance of the blue group may be due to the inadequate contrast between blue and grey stimuli to stomatopod colour vision. Furthermore, the agreement between the CD calculated with the multi-dichromatic processing model and behavioural tests reinforces the idea of a multi-dichromatic processing system in stomatopod colour vision.

#### Possible innate behaviours toward blue stimuli

An alternative explanation for the distinct performance in the blue group may be the stomatopods’ innate response to blue stimuli, potentially influencing the choice tests. Stomatopods inhabit environments rich in spectral and polarisation information as they possess a complex visual system evolved for fitness optimization. Certain signals are hardwired to elicit specific behaviours, aiding in conflict avoidance or enhancing reproductive success. Bok et al. (2018) discovered that *H. trispinosa* can distinguish between UVA and UVB light, with the species displaying an aversive response specifically to UVB. This suggests that UV signals may play a role in species recognition or act as a warning signal in stomatopods. Similarly, Gagnon et al. (2015) found that circular polarisation signals serve as burrow occupation cues in another stomatopod, *Gonodactylaceus falcatus*. Their study showed that *G. falcatus* avoids burrows reflecting circular polarised light, indicating that these signals may help prevent territorial conflicts during burrow searches in some stomatopods.

The maxillipeds of some stomatopods, especially in the genus *Haptosquilla* (How et al., 2014), commonly reflect blue signals, sometimes with strong polarisation (Fig. 9B). Chiou et al. (2011) revealed that the blue-polarised reflection from *H. trispinosa* served as mating signals. Female *H. trispinosa* showed a preference for mating with males possessing blue-polarised maxillipeds, while it took longer for females to accept control males, whose maxillipeds were covered with black paint. These findings imply that *H. trispinosa* may associate blue cues strongly with species recognition, perhaps making it a unfavoured signal for food choice.

In Streets et al. (2022), naive tests revealed intriguing behaviours in *H. trispinosa* regarding their responses to various stimuli. While they did not exhibit aversion to saturated blue stimuli compared with saturated red, orange and green ones, they did display a preference for low-saturated blue when presented with both saturated and unsaturated blue stimuli. Similarly, in naive tests comparing saturated blue with grey, stomatopods favoured grey over saturated blue. These findings indicated that saturated blue stimuli may elicit innate avoidance behaviours, particularly when presented alongside stimuli with low saturation levels, such as unsaturated blue or grey stimuli. However, the interplay of polarisation, colour and brightness information complicates determination of whether the reflectance of the maxillipeds in *H. trispinosa* resembles unsaturated or saturated blue in their visual system. Nonetheless, given their evident avoidance of saturated blue stimuli, it cannot be ruled out that their innate behaviours may contribute to the learning challenges and poor performance observed in the blue group.

### Information processing in stomatopods colour vision

Comparing the visual system of stomatopods with other invertebrates sheds light on the processing mechanisms of the multi-dichromatic system. In many insects, photoreceptors responsible for colour vision typically terminate in the medulla, the second optic lobe, where spectral information is pooled and analysed (Paulk et al., 2008, 2009; Schnaitmann et al., 2018, 2020). For instance, *Drosophila melanogaster* has two types ommatidia: ‘pale’ and ‘yellow’. The R7 and R8 in pale ommatidia detect short-UV and blue light, while those in yellow ommatidia respond to long-UV and green light (Schnaitmann et al., 2018, 2020). These photoreceptors deliver spectral information directly to the medulla, where opponent processing occurs through reciprocal inhibition between R7 and R8 terminals of each ommatidium, with additional opponent mechanisms comparing UV information between ommatidia and integrating spectral and spatial data. The dual colour-opponent system of pale and yellow ommatidia in *D. melanogaster* demonstrates how dichromatic pathways can operate within a polychromatic visual system.

Stomatopods likely possess a similar dichromatic system to that in *D. melanogaster*. However, unlike most insects, their photoreceptors (R1–R7) for colour vision at wavelengths of 400–750 nm terminate in the lamina rather than the medulla. This suggests that colour-opponent processing in stomatopods might occur in the lamina, where rows 1–4 may function as four opponent systems, streamlining polychromatic information into distinct channels before it reaches the medulla. Additionally, stomatopods exhibit a unique midband processing pathway in the medulla (Thoen et al., 2017a, 2018), which exclusively receives projections from midband lamina neurons and is separated from the neuron representations of hemisphere regions. These findings support the hypothesis that their spectral information might be primarily processed in the lamina, with the medulla serving as a relay station without complex processing, consistent with the concept of binning processing.

Although early colour-opponent processing in the lamina is uncommon, it has been identified in the visual system of the butterfly *Papilio Xuthus*, which possesses up to eight classes of spectrally distinct photoreceptors, making it the only other animal with comparable retinal complexity to stomatopods (Arikawa et al., 1987; Arikawa, 2003; Koshitaka et al., 2008; Marshall and Arikawa, 2014). Spectral opponency in *P. xuthus* occurs at the lamina via inter-photoreceptor synapses (Chen et al., 2013, 2019, 2020). This early stage of visual processing is thought to streamline spectral signals, leading to more effective information processing. Given that stomatopods have even more spectral channels and also process complicated polarisation vision, they likely face a similar challenge of managing complex retinal data. It is plausible that, like *P. xuthus*, stomatopods use early-stage spectral processing in the lamina to reduce computational load and extract critical information efficiently. Further neuroanatomical and electrophysiological studies are needed to better understand stomatopod spectral processing pathways.

While our study primarily focused on colour-opponent and multi-dichromatic systems within R1–R7 for visible wavelengths (400–750 nm), it is important to note that these systems are not confined to dichromatic processing in R1–R7 alone. The UV-sensitive R8 cells, despite their axons terminating in the medulla, have synapses in the lamina that may integrate UV signals with other R1–R7 cells at this stage (Thoen et al., 2018). Intriguingly, these synaptic arborisations in the lamina are unique to R8 cells in rows 1–4 (associated with colour vision), whereas those for polarisation vision are relatively minimal. This suggests that UV information from R8 cells may be involved in spectral processing in the lamina. As previously mentioned, stomatopods can distinguish between different UV lights (Bok et al., 2018), hinting at the possibility of UV-opponency between R8 signals from different rows. However, whether and how UV signals are integrated into the colour opponency system in the lamina or processed independently in the medulla remains unknown and requires further investigation.

## Supplementary Material

10.1242/jexbio.247952_sup1 Supplementary information
