# Supplementary material for: Behavioural evidence of spectral opponent processing in the visual system of stomatopod crustaceans
Source: J Exp Biol. 2025 Jan 8;228(1):jeb247952. doi: 10.1242/jeb.247952 (PMC11744319; doi:10.1242/jeb.247952)
Supplement: Supplementary information [file jexbio-228-247952-s1.pdf]

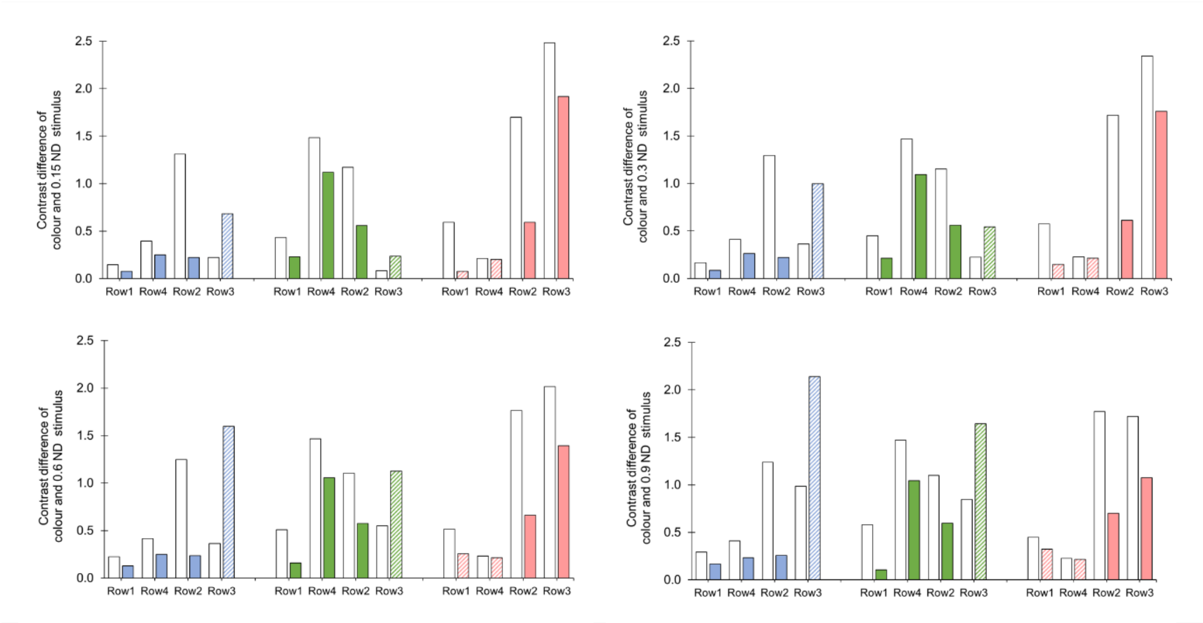

Fig. S1. Relative contrast difference (CD) of colour and grey stimuli.

The figure illustrates the CD between colour and different grey stimuli (0.15 ND, 0.3 ND, 0.6 ND, 0.9 ND) as determined by spectral rows R1-4 under both natural light and coloured illuminations. The white bars represent the CD under natural light, while the coloured bars depict the conditions under coloured illumination. The filled colour bar indicates the activated spectral row. The coloured bars with lines represent the rows that were not activated under the corresponding coloured illumination.

Supplementary Appendix

Table S1. Pseudo-randomised stimulus combination. A total of 36 test trials were designed using one colour target at three positions alongside six combinations of grey distractors (0.15/0.3, 0.15/0.6, 0.15/0.9, 0.3/0.6, 0.3/0.9, and 0.6/0.9). The position and order of tests were arranged using a pseudo-random method to prevent the same colour or grey stimulus from appearing in the same position for more than two consecutive trials. The first table lists the combinations of colour and grey stimuli, while the second table provides a summary of the stimulus positions.

Test	Combination			Test	Combination			Test	Combination		
Position	Left	Middle	Right	Position	Left	Middle	Right	Position	Left	Middle	Right
1	C	0.3	0.6	13	0.3	C	0.9	25	0.6	0.3	C
2	0.6	C	0.15	14	0.6	0.9	C	26	C	0.6	0.9
3	0.3	0.9	C	15	C	0.9	0.15	27	0.15	C	0.9
4	0.6	C	0.3	16	0.9	C	0.6	28	C	0.15	0.9
5	C	0.15	0.3	17	C	0.3	0.9	29	0.9	0.15	C
6	0.3	0.6	C	18	0.6	C	0.9	30	0.15	C	0.3
7	C	0.9	0.6	19	C	0.3	0.15	31	0.15	0.9	C
8	0.3	0.15	C	20	0.9	C	0.15	32	C	0.15	0.6
9	C	0.6	0.15	21	0.9	0.3	C	33	0.9	0.6	C
10	0.3	C	0.15	22	C	0.9	0.3	34	C	0.6	0.3
11	0.15	0.3	C	23	0.15	0.6	C	35	0.15	C	0.6
12	0.6	0.15	C	24	0.9	C	0.3	36	0.3	C	0.6

Stimulus	Count (36 tests)		
Position	Left	Middle	Right
Colour	12	12	12
0.15	6	6	6
0.3	6	6	6
0.6	6	6	6
0.9	6	6	6

Journal of Experimental Biology • Supplementary information

Table S2. Effect of grey distractor combinations on choice under different light conditions in Experiment 1. Results of the Generalized Linear Mixed Model (GLMM) analysis, using distractor combination as a fixed factor and individual (ID) as a random factor: Choice ~ Combination + [1 | ID]. The table presents the coefficients and significance values (Probability > |z|) for each combination of grey distractors under both natural light and coloured tent conditions. Three target colour groups—blue, green, and red—are examined, with the corresponding coefficients and p-values listed for each distractor combination.

(A)

Light condition	Distractor combination	Exp1_Group and target colour					
		Blue		Green		Red	
		Coefficient	Pr (>|z|)	Coefficient	Pr (>|z|)	Coefficient	Pr (>|z|)
Natural light	0.15/0.3	1.386	0.215	0.682	0.132	0.747	0.065
	0.15/0.6	-0.981	0.497	0.85	0.274	0.987	0.185
	0.15/0.9	NA	NA	15.281	0.997	-32.461	1.000
	0.3/0.6	-0.134	0.923	0.786	0.267	1.099	0.138
	0.3/0.9	0.000	1.000	0.159	0.846	-0.342	0.654
	0.6/0.9	31.716	1.000	-0.592	0.439	-0.342	0.654
Coloured tent	0.15/0.3	0.000	1.000	1.992	0.001	1.635	0.005
	0.15/0.6	0.000	1.000	-0.318	0.717	-0.526	0.547
	0.15/0.9	-35.539	1.000	-1.705	0.082	-1.613	0.072
	0.3/0.6	-0.916	0.577	-1.373	0.076	-1.091	0.142
	0.3/0.9	0.000	1.000	-1.299	0.115	-0.521	0.516
	0.6/0.9	0.693	0.711	-0.671	0.421	-0.234	0.766

Journal of Experimental Biology • Supplementary information

Table S3. Effect of grey distractor combinations on choice under different light conditions in Experiment 2. Results of the Generalized Linear Mixed Model (GLMM) analysis with distractor combination as a fixed factor and individual (ID) as a random factor: Choice ~ Combination + [1 | ID]. The table presents the coefficients and significance values (Probability > |z|) for each combination of grey distractors under different light conditions. Significant effects were observed in the red group under the double tent, where the combinations 0.15/0.9 and 0.3/0.6 led to incorrect choices, with animals tending to select the darker grey distractors. (B) Summary of wrong choices in the red group under double tent conditions, showing a preference for darker distractors (0.9 and 0.6 ND) in the 0.15/0.9 and 0.3/0.6 combinations.

Light condition	Distractor combination	Exp2_Group and target colour					
		Blue		Green		Red	
		Coefficient	Pr (>|z|)	Coefficient	Pr (>|z|)	Coefficient	Pr (>|z|)
Natural light	0.15/0.3	0.288	0.706	2.67	0.06	0.636	0.360
	0.15/0.6	1.322	0.322	0.045	0.977	-0.102	0.908
	0.15/0.9	-0.288	0.819	16.665	0.991	1.038	0.421
	0.3/0.6	0.405	0.779	-0.883	0.595	0.571	0.590
	0.3/0.9	1.658	0.207	-2.236	0.113	0.015	0.986
	0.6/0.9	0.811	0.558	-2.107	0.185	1.092	0.400
Double tent	0.15/0.3	0.511	0.484	0.223	0.739	1.101	0.063
	0.15/0.6	-2.303	0.077	0.47	0.630	-1.128	0.159
	**0.15/0.9**	-32.63	1.000	0.182	0.872	**-1.827**	***0.049****
	**0.3/0.6**	-0.511	0.68	0.875	0.512	**-2.081**	***0.047****
	0.3/0.9	-2.457	0.058	0.288	0.772	-1.111	0.140
	0.6/0.9	-1.609	0.239	-1.322	0.322	-1.434	0.144
0.3 ND tent	0.15/0.3	1.946	0.069	1.792	0.097		
	0.15/0.6	31.919	1.000	46.798	1.000		
	0.15/0.9	-0.847	0.590	34.762	1.000		
	0.3/0.6	-0.847	0.590	-0.405	0.794		
	0.3/0.9	-0.154	0.919	-0.405	0.762		
	0.6/0.9	-1.253	0.441	32.752	1.000		
0.6 ND tent	0.15/0.3	21.138	0.985	1.386	0.080		
	0.15/0.6	-19.192	0.986	0.811	0.538		
	0.15/0.9	-21.138	0.985	34.992	1.000		
	0.3/0.6	-21.138	0.985	0.000	1.000		
	0.3/0.9	-20.04	0.985	-0.134	0.906		
	0.6/0.9	-20.04	0.985	0.000	1.000		
0.9 ND tent	0.15/0.3	-0.288	0.706	1.946	0.069		
	0.15/0.6	0.981	0.497	-1.54	0.273		
	0.15/0.9	0.981	0.497	-0.56	0.718		
	0.3/0.6	37.132	1.000	-0.56	0.718		
	0.3/0.9	0.000	1.000	31.283	1.000		
	0.6/0.9	0.288	0.797	38.482	1.000		

(A)

Journal of Experimental Biology • Supplementary information

	Distractor Combination	Total wrong choices recorded	Chose 0.15 or 0.3	Chose 0.9 or 0.6
Exp2 Double tent (Red group)	0.15/0.9	6	1	5
	0.3/0.6	5	2	3

(B)

Journal of Experimental Biology • Supplementary information

Table S4. Individual performance in Experiment 1.

	Blue group	Green group	Red group
**Number of primed and trained individuals**	**27**	**20**	**22**
**Number of individuals tested**	**9**	**9**	**8**
	(♂: 5; ♀4)	(♂: 6; ♀3)	(♂: 3; ♀5)
Number of valid natural light trials in the group	69	88	91
Percentage of correct choices (%)	53.62	72.72	74.73
Percentage of no choices (%)	47.72	16.19	13.34
**Number of individuals included in the analysis**	**4**	**8**	**8**
	(♂: 2; ♀2)	(♂: 6; ♀2)	(♂: 3; ♀5)
Trainability (%)	14.81	40.00	36.36
Natural light trials			
Number of valid trials in the group	25	83	91
Percentage of correct choice (%)	76.00	71.08	74.73
Percentage of no choices (%)	44.45	13.54	13.34
Coloured illumination trials			
Number of valid trials in the group	20	107	99
Percentage of correct choice (%)	40	75.70	73.74
Percentage of no choices (%)	66.67	19.55	27.74

Journal of Experimental Biology • Supplementary information

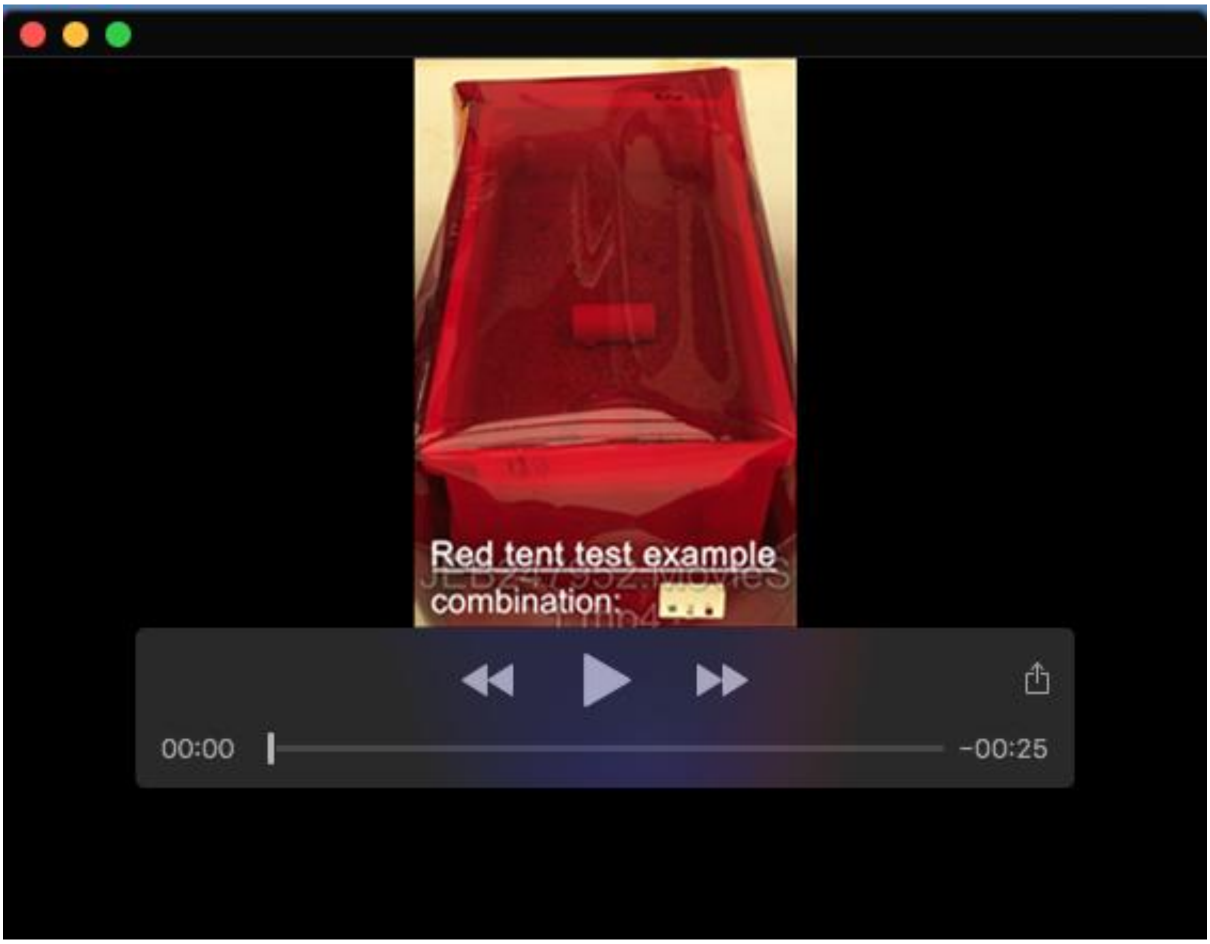

Movie 1. Stomatopod *H. trispinosa* conducting testing trial under coloured illumination. The three choices from left to right were 0.6 ND, 0.3 ND and red stimuli, respectively.

Journal of Experimental Biology • Supplementary information
